# The root of the mystery: the role of long non-coding RNAs in regulating transcription

**DOI:** 10.1093/plphys/kiad434

**Published:** 2023-08-01

**Authors:** Amy Lanctot

**Affiliations:** Assistant Features Editor, Plant Physiology, American Society of Plant Biologists; Cold Spring Harbor Laboratory, Cold Spring Harbor, NY, USA 11724

Gene expression specificity is a core aspect of transcriptional regulation—genes are expressed in specific temporal and spatial patterns to promote distinct developmental and environmental responses. Transcriptomic analyses have elucidated many of these gene expression profiles but have mostly focused on genes that encode for proteins. Far less studied is the role of other transcriptional products, such as long intergenic noncoding RNAs (lincRNAs), in gene expression. In animal systems, lincRNAs have been shown to affect chromatin structure, mRNA splicing and stability, and translation (Statello et al. 2021). In plants, a couple studies have surveyed lincRNAs in the genome, but these studies did not examine the expression specificity of these loci (Liu et al. 2012; Zhao et al. 2018).

In this issue of *Plant Physiology*, Liu et al. (2023) combined more than 100 transcriptomic studies to produce a unified set of nearly 7,000 annotated lincRNA in Arabidopsis. The authors took advantage of this rich dataset to determine various biological attributes of these lincRNAs, such as their conservation across deep evolutionary time, their spatial and condition-specific expression profiles, and their regulation by transcription factor networks. For each locus, the authors determined both the presence of homologs in other plant species, determining how evolutionarily conserved the lincRNA was (Fig. 1A), and the selective pressure on the locus, by evaluating sequence conservation of the lincRNA itself as well as its promoter. Surprisingly, the lincRNAs that were conserved within Brassicaceae species, but not other eudicot or angiosperms, showed the strongest selective pressure, suggesting these Brassicaceae-specific lincRNAs may be functional. This result differs from animal systems, where the most conserved lincRNA loci are under the strongest selective pressure and consequently are thought to have the most functional relevance (Sarropoulos et al. 2019).

**Figure 1.**
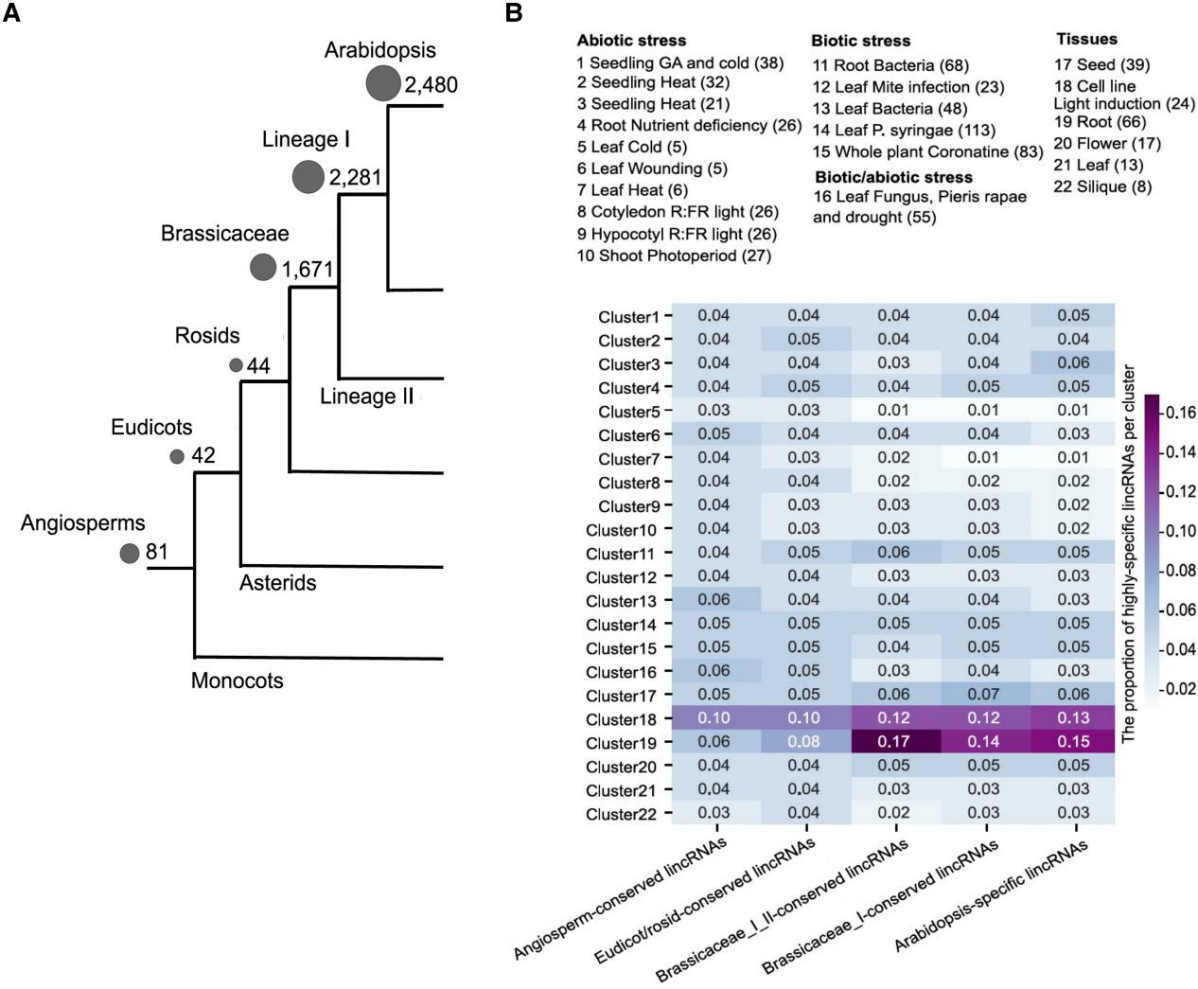
LincRNAs in Arabidopsis are conserved in angiosperms and expressed in diverse profiles. **A)** Categorization of lincRNAs by extent of conservation through evolutionary time. The number of lincRNAs conserved in each evolutionary category is indicated by the gray circles. **B)** Expression profiles of lincRNAs by tissue- and condition-specific clusters. Cluster identities are annotated at the top. The heat map indicates the extent of specificity in lincRNA expression in each cluster, with lincRNAs grouped in columns by their evolutionary conservation. Adapted from Liu et al. (2023).

The authors went on to classify the lincRNAs by their expression profiles, using tissue- and condition-specific transcriptomic data. The lincRNAs grouped in distinct clusters with specific expression profiles and in fact were more specific in their expression patterns than protein-coding genes. The root-expressed cluster showed the most specificity, suggesting that lincRNAs may play an important role in transcriptional regulation of root development (Fig. 1B). The authors then determined potential regulation of lincRNA expression by transcription factors (TFs) by integrating ChIP-seq data into their analysis. They found that lincRNAs expressed in roots were enriched for regulation by several TFs known to play a role in root development (Hawker and Bowman 2004; Li et al. 2022), such as KANADI1 and PHYTOCHROME-INTERACTING FACTOR 4, and validated these interactions by quantitative PCR analysis of TF mutants and DNA affinity purification (DAP)-seq analysis of TF binding. They also used assay for transposase-accesible chromatin (ATAC)-seq data to confirm that the TF-binding peaks associated with lincRNA transcription were in accessible chromatin regions in the root and found that indeed 93% of these peaks were associated with open chromatin, further suggesting that these regulatory relationships are occurring in vivo. Interestingly, the lincRNAs regulated by these TFs were mostly conserved within the Brassicaceae and consequently under strong selective pressure, further indicating their potential functionality (Fig. 1B).

Using a variety of genomic datasets, Liu et al. identified thousands of Arabidopsis lincRNAs and determined their evolutionary conservation and selection signatures, tissue-specific expression profiles, chromatin status, and potential regulation by TFs. By focusing on specificity in lincRNA transcriptional profiles, the authors were able to contextualize their annotations and generate hypotheses as to the function of these lincRNAs. This dataset can serve as a resource for researchers interested in targeting lincRNA loci for CRISPR-based disruption, allowing rapid and potentially high-throughput functional analysis of lincRNAs’ role in development. Recent advancements in tissue-specific and inducible CRISPR systems can expand the complex genetics that can be revealed through such precision editing approaches (Pan et al. 2022). The authors found that the most highly expressed lincRNAs were also the most deeply conserved, but these deeply conserved lincRNAs were not under the highest selective pressure. This result may indicate that lincRNAs are more prominent in their role in plant developmental regulation, especially in the Brassicaceae, as more family-specific lincRNA are likely to be functional. These Brassicaceae-specific lincRNAs would be interesting to further explore, as perhaps they regulate Brassicaceae-specific developmental innovations such as glucosinolate production (Doghri et al. 2021).

The authors used an innovative approach to determine relationships between TF-binding and lincRNA expression and found that many evolutionarily constrained lincRNAs were regulated by known gene regulatory networks. Interestingly, lincRNA promoters did not contain the conserved sequences that are often hallmarks of TF binding, suggesting that, unlike for protein-coding genes, regulation of these loci may not rely on sequence conservation. Further research could analyze the mechanism by which TFs regulate lincRNA expression, which may rely on chromatin accessibility or positional information (Mohammadin et al. 2015). These gene regulatory network analyses also revealed a complex regulon of root-expressed lincRNA by known root developmental TFs. This model of correlating lincRNA expression profiles with the binding of TFs of known function is an excellent approach to generate hypotheses to the role uncharacterized lincRNAs play in development. This article represents both a rich resource for future experimental work and an impressive integration of genomic data that shapes our understanding of transcriptional regulation by lincRNAs.

## Dive Curated Terms

The following phenotypic, genotypic, and functional terms are of significance to the work described in this paper:

cluster CHEBI: CHEBI:33731
